# Umbilical arterial catheter duration as risk factor for Bell's Stage III necrotizing enterocolitis in preterm neonates

**DOI:** 10.1002/jpr3.12081

**Published:** 2024-05-20

**Authors:** Renjini Lalitha, Matthew Hicks, Mosarrat Qureshi, Kumar Kumaran

**Affiliations:** ^1^ Department of Pediatrics, Division of Neonatal‐Perinatal Medicine University of Western Ontario London Ontario Canada; ^2^ Department of Pediatrics Division of Neonatal‐Perinatal Medicine Edmonton Alberta Canada

**Keywords:** necrotizing enterocolitis, neonates, preterm, risk factors, umbilical arterial catheter

## Abstract

**Objectives:**

To explore risk factors for Stage‐III necrotizing enterocolitis (NEC‐III) in preterm neonates.

**Methods:**

This was a retrospective case‐control study of neonates born <33 weeks gestational age (GA) who were admitted to a tertiary neonatal intensive care unit, between 2015 and 2018. NEC‐III cases were compared with Stage‐II NEC (NEC‐II) and non‐NEC controls. Two to four non‐NEC controls were matched by GA ± 1 week and date of birth ± 3 months, to one NEC‐III case. Univariate and multivariate analyses were used to examine risk factors for NEC‐III.

**Results:**

Of 1360 neonates born <33 weeks, 71 (5.2%) had NEC‐II and above, with 46% being NEC‐III. Mean age of onset of NEC‐III was 13.7 days versus 23.9 days for NEC‐II (*p* = 0.01). Neonates with NEC‐III were of lower GA (NEC‐III 25.4 weeks, NEC‐II 27.3 weeks, and non‐NEC 26 weeks; *p* = 0.0008) and had higher Score for Neonatal Acute Physiology Perinatal Extension‐II scores (NEC‐III 47.5, NEC‐II 28.4 and non‐NEC 37, *p* = 0.003). Multivariate analysis showed duration of umbilical arterial catheter (UAC) >5 days was significantly associated with the development of NEC‐III with adjusted odds ratio (AOR) 3.8; 95% confidence interval (CI) (1.05–13.66) for NEC‐III versus non‐NEC and AOR 5.57; 95% CI (1.65–18.73), *p* = 0.006 for NEC‐III versus NEC‐II. Rupture of membranes (ROM) >1 week was associated with NEC‐III (AOR 6.93; 95% CI [1.56–30.69] vs. non‐NEC and AOR 11.74; 95% CI [1.14–120.34] vs. NEC‐II).

**Conclusion:**

The increased association of NEC‐III with duration of UAC and ROM could be further examined in prospective studies, and an upper limit for UAC duration could be considered in NEC prevention bundles.

## Abstract (highlights)

**What is Known:**

• Stage‐III necrotizing enterocolitis (NEC‐III) has high mortality and morbidity among all types of NEC. Risk factors for the development of NEC in general have been documented in many studies but those for NEC‐III in particular remain unexplored.

**What is New:**

• NEC‐III occurs earlier compared to NEC‐II suggesting a unique predisposing factor to its development.
• Duration of umbilical artery catheterization (UAC) greater than 5 days was associated with the development of NEC‐III.
• An upper limit for the duration of UAC might be warranted for the prevention of NEC.

## 1 INTRODUCTION

Necrotizing enterocolitis (NEC) is a serious intestinal inflammatory disease in neonates first described in 1965 by Mizrahi et al.^1^ The disease is characterized by inflammation and injury of the gut wall barrier that may advance to necrosis and potentially, perforation of the gut.^2^, ^3^ NEC occurs in 5%–10% of very low birth weight neonates (VLBW).^4^, ^5^

NEC is categorized into three stages based on modified Bell's staging criteria, namely, Stage I—suspected NEC, Stage II proven NEC, and Stage III Advanced NEC based on severity of clinical signs and symptoms, radiological findings and laboratory abnormalities as described in Appendix 1.^6^, ^7^ The clinical presentation of NEC can be highly variable mimicking sepsis but the presence of pneumatosis intestinalis and/or portal venous gas on X‐rays or necrotic bowel at the time of surgery helps to differentiate NEC stage II or above from sepsis. While stage I NEC includes patients with mildest symptoms, radiological signs of pneumatosis intestinalis confirm Stage II NEC. Stage III NEC (NEC‐III) is the severe form and reported to comprise 20%–40% of all cases. There is a high mortality rate with significant morbidity in survivors.^8^, ^9^ NEC‐III not only shows all the clinical and radiological signs of stage I and II NEC but also deterioration of vital signs, presence of shock, coagulopathies, bowel necrosis, and pneumoperitoneum. Several risk factors are associated with the development of NEC.^4^, ^10^, ^11^, ^12^ The most consistent epidemiologic risk factors for NEC have been identified as prematurity and enteral feeding practices.^13^, ^14^

More recently, Sulemanji and group, through their observational study found the presence of an umbilical venous catheter (UVC) into the portal vein or ductus venosus was associated with an increased incidence of NEC in VLBW neonates.^15^ While there is some evidence showing impaired splanchnic circulation with umbilical artery catheterization (UAC),^16^, ^17^ there are conflicting results on its presence or tip position and associated development of NEC.^18^, ^19^ Although Thompson et al. looked at risk factors for NEC‐totalis (defined as greater than 80% intestinal necrosis),^20^ there have been no studies looking at risk factors for NEC‐III in general.

### 1.1 Study rationale

NEC‐III has a high case fatality rate and is associated with prolonged hospital stay, total parenteral nutrition dependence, liver dysfunction, neurodevelopmental disabilities, growth failure, short bowel syndrome, intestinal scarring, and need for surgical intervention.^8^, ^9^ To date there have been no study comparisons assessing risk factors for the development of NEC‐III, and specifically, its association with duration of umbilical line placement. The aim of this exploratory study is to develop a specific hypothesis for the prediction of NEC‐III development in preterm neonates, which could be tested in future research.

### 1.2 Objectives

1. To assess the risk factors associated with the development of NEC‐III in preterm neonates born less than 33 weeks gestational age (GA).
2. To investigate the association of the presence and duration of umbilical catheterization with the development of NEC‐III in preterm neonates born less than 33 weeks GA.

### 1.3 Definitions

1. NEC was scored according to the following criteria: (a) definite pneumatosis (air within the bowel wall) or portal/hepatic gas as diagnosed by x‐ray or ultrasonography or (b) surgical or autopsy diagnosis of NEC. Diagnosis of “suspected NEC” or x‐rays showing pneumoperitoneum without pneumatosis will not be classified as NEC. NEC cases were staged as per Modified Bell's staging criteria (Supporting Information S1: Appendix S1).
2. Vasoactive agents are agents that increase myocardial contractility (inotropes) and/or increase vascular tone like vasopressors. The Vasoactive Inotrope score quantifies the amount of cardiovascular therapy using a weighted sum of all the inotropic and vasopressor agents (Supporting Information S1: Appendix S2).

## 2 METHODS

This was a single‐center retrospective case‐control study conducted at a level three neonatal intensive care unit (NICU) in West Canada between January 2015 and December 2018 using a combination of neonatal database and chart review This center is one of the largest tertiary perinatal centers in Canada with a 69 bed NICU that receives approximately 1300 admissions annually. Neonates born less than 33 weeks who were managed as NEC‐III during the study period formed the case group and those with NEC‐II and without NEC formed the two control groups. The NEC‐III cases were compared with NEC‐II (1NEC‐III: 1NEC‐II) and with non‐NEC controls (1 NEC‐III case: 2–4 non‐NEC) that were matched for GA ± 1 week, and date of birth within 3 months. Neonates with lethal congenital malformations, chromosomal/genetic abnormalities, congenital heart defects (except for patent ductus arteriosus, ventricular septal defects, and atrial septal defects), and intestinal anomalies were excluded. All cases of NEC that were managed in the NICU were reviewed in detail by the local NEC working group committee consisting of neonatologists, neonatal fellows, radiologists, nurse practitioners, nurses, nutritionists, and clinical educators. The diagnosis of NEC and its stages were confirmed by this committee, using Modified Bell's staging criteria (Supporting Information S1: Appendix S1).

### 2.1 Data collection

We conducted extensive chart and database review for maternal and neonatal demographics, and perinatal data such as GA, birth weight, sex, antenatal steroid/magnesium sulfate, pregnancy‐related morbidities, mode of delivery, Apgar score, surfactant use, and cord gases. Data on feeding, vasoactive agents, respiratory support, sepsis, presence of patent ductus arteriosus (PDA), need for nonsteroidal anti‐inflammatory drugs (NSAIDS) for PDA treatment, presence and duration of umbilical lines, and presence of malpositioned umbilical lines before diagnosis of NEC in both NEC‐III and NEC‐II groups or within 60 days of life for non‐NEC group were also collected.

### 2.2 Statistical analysis

Given the retrospective and exploratory nature of the study, an a priori sample size calculation was not performed and a sample of convenience was used. All statistical analyses were performed with Intercooled Stata Version 15.0 (StataCorp). All data were anonymized for analysis. Groups within the study were compared using descriptive statistical methods. Maternal and neonatal characteristics were compared between groups using Pearson's *χ* ^2^ and Fisher's exact tests for categorical variables and one‐way analysis of variance (for comparing three groups—NEC‐III, NEC‐II, and non‐NEC) or Student's *t* test for comparing continuous variables. Histograms and boxplots were used to assess underlying assumptions of normal distribution and normality of variances. Multivariate logistic regression models were built to identify risk factors associated with the development of NEC‐III compared to NEC‐II and controls using a forward and backward stepwise selection approach and variables were included if they had a *p* value less than 0.2 in bivariate analysis. Associations between variables and outcomes were expressed as adjusted odds ratios (AORs) and 95% confidence intervals (CIs). A *p* value < 0.05 was considered significant. Adjustments for multiple comparisons were not made given the exploratory nature of this work. Unmatched analyses are presented. This study was approved by the Northern Alberta Clinical Trials and Research Center and the Human Research Ethics Office at the University of Alberta.

## 3 RESULTS

**Figure 1:**
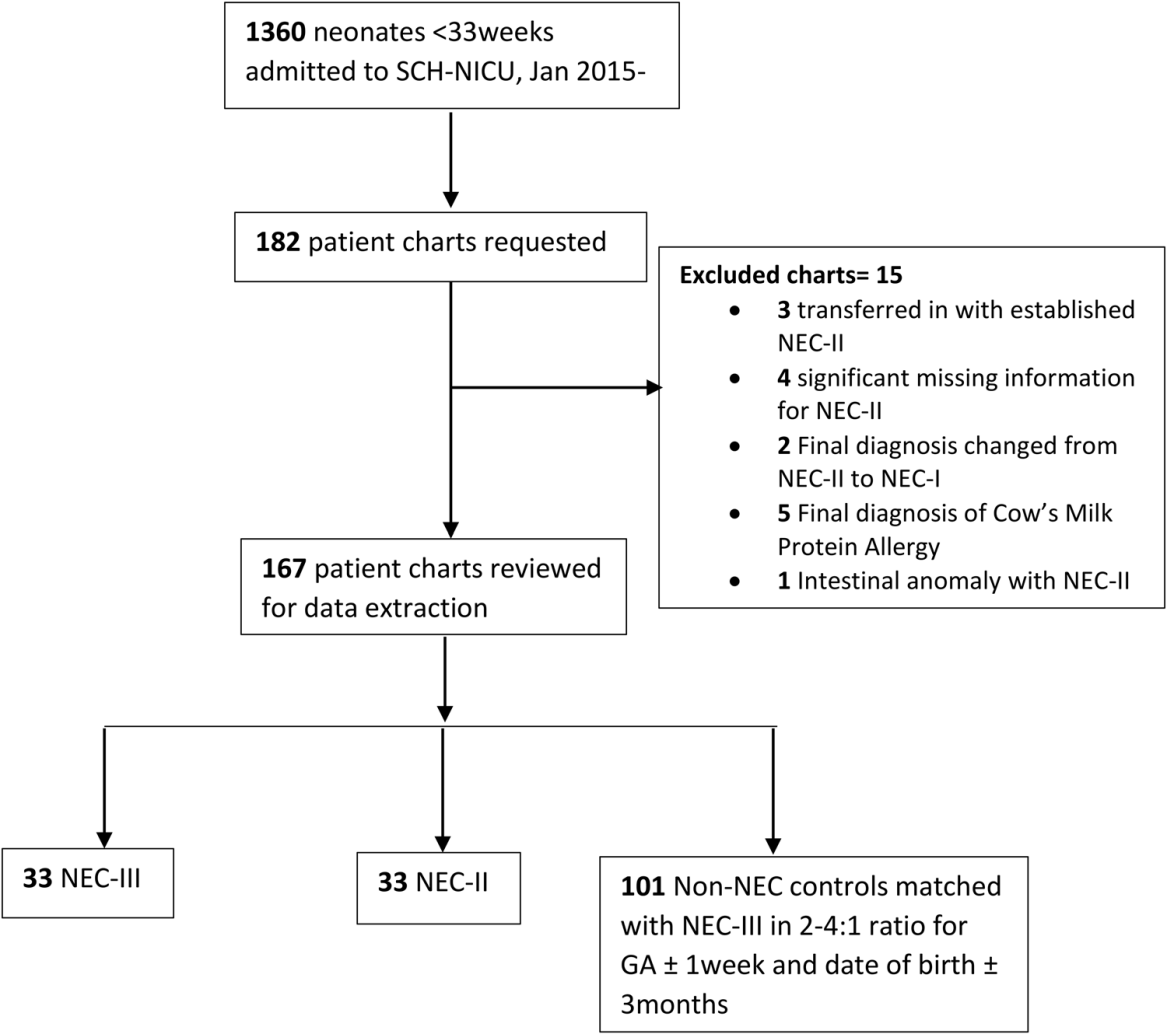
Study flow diagram. GA, gestational age; NEC‐I, stage‐I necrotizing enterocolitis; NEC‐II, stage‐II necrotizing enterocolitis; NEC‐III, stage‐III necrotizing enterocolitis; NICU, neonatal intensive care unit; SCH, Stollery Philip C. Etches Hospital.

A total of 1360 preterm neonates born less than 33 weeks were admitted to our center during the 4‐year study period. The incidence of NEC‐II and NEC‐III was 5.2% in VLBW, with 46% of cases meeting the criteria for NEC‐III (Figure 1). Neonates with NEC‐III were of lower GA (25.4 ± 2.3 weeks) compared to NEC‐II (27.3 ± 2.7 weeks) (*p* = 0.0003, data not shown). Average age of onset of NEC‐III was 13.7 (12.2 standard deviation [SD]) days versus 23.9 (18.8 SD) days for NEC‐II (*p* = 0.01).

Table 1 compares the demographic, clinical, and biochemical characteristics of the NEC‐III, NEC‐II, and non‐NEC groups. Severity of illness was defined using the Score for Neonatal Acute Physiology Perinatal Extension‐II (SNAPPE‐II score). Neonates with NEC‐III had a higher severity of illness (SNAPPE‐II 47.5; SD 25.1) than NEC‐II infants (SNAPPE‐II 28.4; SD 19.5) and non‐NEC infants (SNAPPE‐II 37; SD 22.1), (*p* = 0.003). Infants who developed NEC‐III were on vasoactive agents longer (7.5 days vs. 3.2 days and 4.2 days for NEC‐III, NEC‐II, and non‐NEC, respectively; *p* = 0.09), although this was not statistically significant. There were no significant differences in the baseline lactate/maximum metabolic deficits after birth, need for normal saline bolus, and peak vasoactive inotrope scores within the first 3 days of life between groups. Rates of antenatal steroids exposure and Apgar scores <7 at 10 min were not statistically significant among study groups.

**Table 1:** Baseline characteristics comparing NEC‐III, NEC‐II, and non‐NEC groups.

Variable	NEC III (*n* = 33)	NEC II (*n* = 33)	Non‐NEC (*n* = 101)	*p* Value
Gestational age in weeks, mean (SD)	25.4 (2.3)	27.3 (2.7)	26.0 (1.9)	0.0008
Birth weight in grams, mean (SD)	838.3 (291.5)	1003.6 (350.5)	909.1 (289.7)	0.0847
Male sex, *n* (%)	18 (54.5)	22 (66.7)	64 (63.4)	0.560
Outborn births, *n* (%)	4 (12.9)	17.1 (6)	10.9 (1)	0.629
C‐section, *n* (%)	18 (54.5)	18 (54.5)	61 (60.4)	0.651
Antenatal steroids—yes, *n* (%)	27 (81.8)	31 (93.9)	96 (95.0)	0.062
ROM >24 h, *n* (%)	17 (51.5)	29 (87.9)	65 (64)	0.066
ROM >1 week, *n* (%)	7 (21.9)	1 (3.1)	12 (12.1)	0.124
Chorioamnionitis—yes, *n* (%)	7 (21.2)	5 (15.2)	34 (33.8)	0.079
PIH—yes, *n* (%)	1 (3.0)	11 (33.3)	11 (10.9)	0.003
IUGR—yes, *n* (%)	1 (3)	5 (15.1)	2 (2)	0.012
Normal UA dopplers, *n* (%)	27 (81.8)	28 (84.8)	88 (87.1)	0.185
AEDF UA dopplers, *n* (%)	0	5 (15.2)	5 (5)	
REDF UA dopplers, *n* (%)	1 (3)	1 (3)	5 (5)	
Apgar score <7 at 10 min, *n* (%)	11 (33.3)	4 (12.1)	17 (16.8)	0.069
DCC, *n* (%)	16 (49.5)	17 (51.5)	51 (50.5)	0.9721
SNAPPE II, mean (SD)	47.5 (25.1)	28.4 (19.5)	37.0 (22.1)	0.0031
First blood lactate level within 12 h after birth, mean (SD)	3.8 (3.3)	3.9 (2.4)	3.8 (2.7)	0.9888
Max BE in first 12 h after birth, mean (SD)	−7.0 (3.6)	−5.3 (2.2)	−5.6 (3.0)	0.1190
NS bolus within first 72 h of life—no, *n* (%)	15 (45.5)	21 (63.6)	54 (53.5)	0.701
Vasoactive agents within first 72 h of life—yes, *n* (%)	15 (45.5)	12 (36.4)	44 (43.6)	0.428
Days on vasoactive agent, mean (SD)	7.5 (8.0)	3.2 (2.0)	4.2 (5.0)	0.0886
Peak VIS, mean (SD)	6.3 (8.4)	3.8 (7.6)	4.2 (6.3)	0.2848
Postnatal steroids—yes, *n* (%)	0.0 (0)	3 (9.1)	14 (13.9)	0.093
Indomethacin prophylaxis—yes, *n* (%)	6 (18.2)	4 (12.1)	9 (8.9)	0.261
PDA—yes, *n* (%)	20 (60.6)	20 (60.6)	59 (58.4)	0.958
Abdominal aorta Doppler normal, *n* (%)	11 (64.7)	14 (73.7)	40 (63.5)	0.491
Number of courses for PDA treatment, mean (SD)	0.74 (0.86)	0.54 (0.89)	0.73 (1.04)	0.5902
EONS, *n* (%)	0.0 (0)	2 (6.1)	1.0 (1)	<0.001
LONS, *n* (%)	9 (27.3)	7 (21.2)	14 (13.9)	0.138
Days of antibiotics, mean (SD)^c^	5.2 (3.7)	5.4 (4.4)	14.8 (14.1)	<0.0001
PRBC—yes, *n* (%)	19 (57.6)	15 (45.5)	61 (60.4)	0.202
Number of PRBCs, mean (SD)	1.4 (1.6)	0.9 (1.4)	1.8 (2.0)	0.0829
First enteral feed in hours, mean (SD)^a^	10.1 (4.4)	9.6 (5.4)	11.3 (6.0)	0.4187
First enteral feed in days, mean (SD)^b^	2.1 (1.9)	2.8 (4.2)	2.0 (1.9)	0.6242
Never achieved full feeds, *n* (%)	20 (60.6)	12 (36.4)	0 (0)	<0.001
Days on TPN, mean (SD)^c^	10.4 (6.5)	12.9 (9.3)	18.0 (10.4)	0.0004

*Note*: One‐way analysis of variance reported for continuous variables and Fisher's exact test for categorical variables.

Abbreviations: AEDF, absent end diastolic flow; BE, base excess; DCC, delayed cord clamping; EONS, early onset neonatal sepsis; HCO_2_, bicarbonate; IUGR, intrauterine growth retardation; LONS, late‐onset neonatal sepsis; MgSO_4_, magnesium sulfate; NEC‐II, necrotizing enterocolitis stage II; NEC‐III, necrotizing enterocolitis stage III; non‐NEC, non‐necrotizing enterocolitis; NS, normal saline; PDA, patent ductus arteriosus; PIH, pregnancy‐induced hypertension; PRBC, packed red blood cell; REDF, reverse end diastolic flow; ROM, rupture of membrane; SD, standard deviation; SNAPPE II, Score for Neonatal Acute Physiology Perinatal Extension‐II; TPN, total parenteral nutrition; UA, umbilical artery; VIS, vasoactive inotrope score.

a First feed received within first 24 h of life.

^b^ First feed received after 24 h of age.

^c^ Before NEC onset in NEC‐III and NEC‐II groups and within the first 60 days in non‐NEC group.

Table 1 also compares feeding characteristics between the groups. The timing of first enteral feeds was not statistically different between the groups. The majority of neonates with NEC‐III never achieved full enteral feeds before the development of NEC (60.6% in NEC‐III vs. 36.3% in NEC‐II, *p* ≤ 0.0001). Among those who achieved full enteral feeds, the mean days to full feeds did not differ between the groups.

The presence, position, and duration of both umbilical lines were analyzed between NEC‐III, NEC‐II, and non‐NEC groups as shown in Table 2. The rate of UAC insertion after birth was significantly higher in NEC‐III (87.1%) than NEC‐II (55.9%) and non‐NEC (72.3%, *p* = 0.02). In addition, UAC remained in place longer than 5 days in NEC‐III compared to NEC‐II and non‐NEC neonates (NEC‐III 75%, NEC‐II 36.4%, and non‐NEC 47%, *p* = 0.007). X‐ray confirmed optimal UAC and UVC positions were not statistically different between the groups. We further examined the collinearity between UAC days and SNAPPE‐II score in all the study neonates. SNAPPE‐II scores were significantly higher at 48.1 (SD 18.6) in neonates who had UAC in place for >5 days compared with infants with UAC in place for ≤5 days at 25.8 (SD 21.3), *p* ≤ 0.0001. Specifically, among neonates who developed NEC, SNAPPE‐II scores were also significantly higher in those who had UAC in place for >5 days as opposed to those with UAC in place for ≤5 days (51 [SD 18.6] vs. 20.1 [SD 18.9]).

**Table 2:** Comparison of umbilical catheters between NEC‐III, NEC‐II, and non‐NEC groups.

Variable	NEC‐III (*n* = 33)	NEC‐II (*n* = 33)	Non‐NEC (*n* = 101)	*p* Value
Presence of UAC—yes, *n* (%)	27 (87.1)	19 (55.9)	73 (72.3)	0.020
High UAC, *n* (%)	23 (92)	17 (89.5)	68 (95.8)	0.467
Days of UAC,^a^ mean (SD)	8.4 (3.0)	7.1 (3.5)	7.3 (3.6)	0.3599
UAC longer than 5 days,^b^ *n* (%)	21 (75)	12 (36.4)	47 (47)	0.007
Central UVC, *n* (%)	14 (50)	19 (61.3)	49 (50)	0.552
Days of UVC, mean (SD)	7.2 (3.4)	8.8 (4.2)	7.4 (4.1)	0.1749

Abbreviations: NEC‐III, necrotizing enterocolitis stage III; NEC‐II, necrotizing enterocolitis stage II; non‐NEC, non‐necrotizing enterocolitis; UAC, umbilical arterial catheter; UVC, umbilical venous catheter.

a If a UAC was successfully placed.

b If a UAC was present for 5 days or longer.

Multivariate logistic regression analyses were performed to assess factors that were independently associated with NEC‐III development. For this analysis, we created models that included all variables that were *p* < 0.2 in bivariate analysis and performed logistic regression analysis. Most variables including SNAPPE‐II scores did not remain in the final parsimonious regression model. After adjustment for known confounders, length of UAC greater than 5 days had 5.57 (1.65–18.73) and 3.8 (1.05–13.66) odds of developing NEC‐III compared to NEC‐II cases (*p* = 0.006) and non‐NEC controls (0.041), respectively (Table 3).

**Table 3:** Multivariate analysis comparing NEC‐III to NEC‐II and non‐NEC.

	NEC‐III versus NEC‐II		NEC‐III versus non‐NEC	
Variable	Adjusted OR (95% CI)	*p* Value	Adjusted OR (95% CI)	*p* Value
UAC in place for >5 days	5.57 (1.65–18.73)	0.006	3.8 (1.05–13.66)	0.041
Rupture of membranes >1 week	11.74 (1.14–120.34)	0.038	6.93 (1.56–30.69)	0.041

Abbreviations: CI, confidence interval; NEC‐III, necrotizing enterocolitis stage III; NEC‐II, necrotizing enterocolitis stage II; non‐NEC, non‐necrotizing enterocolitis; OR, odds ratio; UAC, umbilical arterial catheter.

## 4 DISCUSSION

In this retrospective case‐control study, we investigated the risk factors for NEC‐III using Bell's criteria in preterm neonates born less than 33 weeks. We found duration of UAC over 5 days and rupture of membranes more than 7 days were significantly associated with increased risk for NEC‐III. NEC‐III can have devastating outcomes in preterm neonates making it important for us to understand risk factors and possible preventive interventions. To the best of our knowledge, this is the first study to date that has looked at risk factors in particular the duration of umbilical lines in association with NEC‐III development.

The incidence of NEC‐III was higher in our study than what is reported in the literature.^8^ This could be explained in part by the fact that our unit is one of the largest units in Canada that cares for a large proportion of VLBW neonates. Our unit also functions as a referral center for a wide catchment area. Unlike NEC‐II, NEC‐III presented earlier in our study, by the second week of life, suggesting a unique predisposing development factor other than feeding practices for preterm neonates.

Numerous factors can influence intestinal blood flow in preterm neonates such as type and advancement of enteral feeding, use of vasoactive agents or NSAIDs, presence of PDA, red blood cell transfusion, sepsis, and umbilical catheterization and its position.^16^, ^17^, ^21^, ^22^, ^23^ Our study showed that neonates with NEC‐III were generally sicker than the control group at birth with higher SNAPPE‐II scores and longer duration of vasoactive agents. This can influence regional gut flow through the diving reflex and local vasoconstrictive effects of the agents, respectively.

While vasoactive agent use may be associated with NEC development,^24^, ^25^, ^26^, ^27^ currently there is lack of robust evidence to incriminate one agent over the other.^28^ Longer duration of vasoactive therapy may suggest extensive circulatory impairment requiring vasoactive therapy. This may lead to derangement in intestinal microcirculation resulting in hypoperfusion and intestinal assault, thus increasing the risk for NEC‐III. Neither duration of vasoactive use nor the use of vasoactive agents significantly increased the risk of NEC‐III in our study. Although there was a trend in longer duration of vasoactive agents use in NEC‐III, a larger sample size may be required to demonstrate significance. Additionally, our study did not look into the granularity of agents used. Two common vasoactive agents used in the preterm population in our center were dopamine and epinephrine.

Antibiotic exposure and its increased association with NEC development have been demonstrated through several studies.^29^, ^30^, ^31^, ^32^, ^33^ More recently, metanalysis by Esaiassen et al.^34^ revealed prolonged antibiotic exposure in noninfected preterm neonates increased the incidence of NEC. Our study did not find such an association. On the contrary, we showed that decreased use of antibiotics increased the incidence of NEC in our cohort. This finding is congruent with findings of others^35^, ^36^, ^37^ demonstrating early antibiotic exposure to be protective against NEC development. The postulated mechanism for this protective effect could be due to targeted manipulation of intestinal microbiomes from early antibiotic exposure that are pathogenic for NEC development. This hypothesis suggests that early colonization may serve an important role in maintaining gut health in these preterm neonates.

Umbilical catheterization is a common procedure performed in VLBW neonates. While a UVC helps with delivery of fluids, parenteral nutrition, intravenous medications, and central venous pressure monitoring, a UAC is used for blood sampling and invasive blood pressure monitoring. Rarely, UACs are used for the administration of fluids and nonvasoactive medications when alternative access is not available. A few retrospective and observational studies have shown intestinal disturbances such as bowel distension, ischemia, and NEC associated with umbilical arterial and venous cannulation and its tip position.^22^, ^38^, ^39^, ^40^ Postulated mechanism of UAC causing intestinal disturbances include microthrombi released by UACs, the vasospasm of splanchnic blood vessels, and reduction in the lumen diameter due to the presence of the catheter in the abdominal aorta, which may lead to decreased blood flow.^38^, ^39^, ^40^ Literature on the effects of UACs on the regional intestinal circulation remains controversial.^16^, ^17^, ^41^, ^42^ It has been postulated that malpositioned UVCs lead to portal venous congestion which may cause intestinal injury.^43^ Disturbances in intestinal blood flow due to UAC and UVC may predispose VLBW neonates to the development of NEC when enterally fed.^44^, ^45^

Our study demonstrated for the first time that the presence of an UAC greater than 5 days is associated with the development of NEC II and NEC‐III. We did not observe an association between location of UAC tip and NEC, which is congruent with the existing scientific evidence.^19^ On the other hand, both the presence and the location of UVC tip were not associated with NEC in our study population.

Risk of NEC‐III increased by over 10‐fold and that for NEC‐II by sevenfold when there was prolonged rupture of membranes (over 1 week) in our cohort. This might be due to increased placental inflammatory markers that the fetus is exposed to that may potentially cause disturbances in intestinal blood flow and intestinal injury. Even though clinical chorioamnionitis was not associated with the development of NEC in our cohort, the antenatal inflammatory process is often clinically silent and evident only in histopathology.^46^, ^47^

Our study has several strengths and limitations. Data collection was limited to one individual, which reduces the introduction of bias as the definition of the clinical parameters assessed were consistent for all the study participants. The short study period limits the potential differences in the way information is recorded in the charts as well as potential changes in the standard of care. The short study period limits any significant change in the standard of practice in preterm neonatal care. Although the data collection process in our study was comprehensive, it is limited to a single institution's experience, and therefore affecting the generalizability of the results. The retrospective nature of the data also makes the availability of information limited to those recorded in electronic and paper charts, which may have been confounded by related variables that were not measured in this study.

## 5 CONCLUSION

The presence of an UAC for greater than 5 days and prolonged rupture of membranes greater than 1 week were associated with severe forms of NEC in VLBW neonates in our study. These data support the need for additional prospective studies to examine the effect of UAC catheter duration on NEC development and consideration for institutional guideline development regarding the use and duration of UAC with respect to NEC prevention until further data is available.

## CONFLICT OF INTEREST STATEMENT

The authors declare no conflict of interest.

## Supporting information

Supplementary Appendix S1: Modified Bell staging criteria for necrotizing enterocolitis.
Supplementary Appendix S2: Calculation of Vasoactive Inotrope score (VIS).

## References

[1] Mizrahi A , Barlow O , Berdon W , Blanc WA , Silverman WA . Necrotizing enterocolitis in premature infants. J Pediatr. 1965;66:697‐706. 14271359 10.1016/s0022-3476(65)80003-8

[2] Yajamanyam PK , Rasiah SV , Ewer AK . Necrotizing enterocolitis: current perspectives. Res Rep Neonatol. 2014;4:31‐42.

[3] Good M , Sodhi CP , Hackam DJ . Evidence‐based feeding strategies before and after the development of necrotizing enterocolitis. Expert Rev Clin Immunol. 2014;10:875‐884. 24898361 10.1586/1744666X.2014.913481 PMC4113601

[4] Neu J , Walker WA . Necrotizing enterocolitis. N Engl J Med. 2011;364(3):255‐264. 21247316 10.1056/NEJMra1005408 PMC3628622

[5] Thompson AM , Bizzarro MJ . Necrotizing enterocolitis in newborns: pathogenesis, prevention and management. Drugs. 2008;68(9):1227‐1238. 18547133 10.2165/00003495-200868090-00004

[6] Neu J . Necrotizing enterocolitis. Pediatr Clin North Am. 1996;43(2):409‐432. 8614608 10.1016/S0031-3955(05)70413-2 PMC7127724

[7] Caplan MS , Jilling T . New concepts in necrotizing enterocolitis. Curr Opin Pediatr. 2001;13(2):111‐115. 11317050 10.1097/00008480-200104000-00004

[8] Petty JK , Ziegler MM . Operative strategies for necrotizing enterocolitis: the prevention and treatment of short‐bowel syndrome. Semin Pediatr Surg. 2005;14:191‐198. 16084407 10.1053/j.sempedsurg.2005.05.009

[9] Henry MCW , Moss RL . Surgical therapy for necrotizing enterocolitis: bringing evidence to the bedside. Semin Pediatr Surg. 2005;14:181‐190. 16084406 10.1053/j.sempedsurg.2005.05.007

[10] Mally P , Golombek S , Mishra R , et al. Association of necrotizing enterocolitis with elective packed red blood cell transfusions in stable, growing, premature neonates. Am J Perinatol. 2006;23:451‐458. 17009195 10.1055/s-2006-951300

[11] Musemeche CA , Reynolds M . Necrotizing enterocolitis following intrauterine blood transfusion. J Pediatr Surg. 1991;26:1411‐1412. 1765924 10.1016/0022-3468(91)91050-9

[12] McGrady GA , Retting PJ , Istre GR , Jason JM , Holman RC , Evati BL . An outbreak of necrotizing enterocolitis. Am J Epidemiol. 1987;126:1165‐1172. 3687923 10.1093/oxfordjournals.aje.a114754

[13] Blakely ML , Lally KP , McDonald S , et al. Postoperative outcomes of extremely low birth weight infants with necrotizing enterocolitis or isolated intestinal perforation: a prospective cohort study by the NICHD Neonatal Research Network. Ann Surg. 2005;241:984‐994. 15912048 10.1097/01.sla.0000164181.67862.7f PMC1359076

[14] Bolisetty S , Lui K , Oei J , Wojtulewicz J . A regional study of underlying congenital diseases in term neonates with necrotizing enterocolitis. Acta Paediatr. 2000;89:1226‐1230. 11083380 10.1080/080352500750027619

[15] Sulemanji M , Vakili K , Zurakowski D , Tworetzky W , Fishman SJ , Kim HB . Umbilical venous catheter malposition is associated with necrotizing enterocolitis in premature infants. Neonatology. 2017;111(4):337‐343. 28092913 10.1159/000451022

[16] Kempley ST , Gamsu HR . Randomised trial of umbilical arterial catheter position: Doppler ultrasound findings. Arch Dis Child. 1992;67:855‐859. 1519989 10.1136/adc.67.7_spec_no.855 PMC1590435

[17] Rand T , Weninger M , Kohlhauser C , et al. Effects of umbilical arterial catheterization on mesenteric hemodynamics. Pediatr Radiol. 1996;26:435‐438. 8662058 10.1007/BF01377197

[18] Barrington KJ . Umbilical artery catheters in the newborn: effects of catheter design (end vs. side hole). Cochrane Database Syst Rev. 2000;1999(2):CD000508. 10796378 10.1002/14651858.CD000508 PMC7038650

[19] Barrington KJ . Umbilical artery catheters in the newborn: effects of position of the catheter tip. Cochrane Database Syst Rev. 2000;1999(2):CD000505. 10796375 10.1002/14651858.CD000505 PMC7038652

[20] Thompson A , Bizzarro M , Yu S , Diefenbach K , Simpson BJ , Moss RL . Risk factors for necrotizing enterocolitis totalis: a case‐control study. J Perinatol. 2011;31:730‐738. 21436786 10.1038/jp.2011.18

[21] Lin PW , Stoll BJ . Necrotising enterocolitis. Lancet. 2006;368(9543):1271‐1283. 17027734 10.1016/S0140-6736(06)69525-1

[22] Kliegman RM . Models of the pathogenesis of necrotizing enterocolitis. J Pediatr. 1990;117(1 Pt 2):S2‐S5. 2113944 10.1016/S0022-3476(05)81123-0 PMC7131608

[23] Panigrahi P . Necrotizing enterocolitis: a practical guide to its prevention and management. Pediatr Drugs. 2006;8(3):151‐165. 10.2165/00148581-200608030-00002 16774295

[24] Acunas B , Vatansever Ü , Duran R , Aladag N . 393 risk factors for necrotizing enterocolitis in very low birth weight infants. Pediatr Res. 2005;58:421.

[25] Gephart SM , Spitzer AR , Effken JA , Dodd E , Halpern M , McGrath JM . Discrimination of GutCheck(NEC): a clinical risk index for necrotizing enterocolitis. J Perinatol. 2014;34(6):468‐475. 24651734 10.1038/jp.2014.37 PMC4420242

[26] Youn YA , Kim EK , Kim SY . Necrotizing enterocolitis among very‐low‐birth‐weight infants in Korea. J Korean Med Sci. 2015;30(suppl 1):S75‐S80. 26566361 10.3346/jkms.2015.30.S1.S75 PMC4641067

[27] Lu CY , Liu KF , Qiao GX , Luo Y , Cheng HQ , DU SZ . Risk factors for necrotizing enterocolitis in preterm infants: a meta analysis. Zhongguo Dang Dai Er Ke Za Zhi. 2022;24(8):908‐916. 36036130 10.7499/j.issn.1008-8830.2202085 PMC9425871

[28] Osborn DA , Paradisis M , Evans N . The effect of inotropes on morbidity and mortality in preterm infants with low systemic or organ blood flow. Cochrane Database Syst Rev. 2007;1:24. 10.1002/14651858.CD005090.pub2 PMC8860620 17253539

[29] Torrazza RM , Neu J . The altered gut microbiome and necrotizing enterocolitis. Clin Perinatol. 2013;40:93‐108. 23415266 10.1016/j.clp.2012.12.009 PMC3589666

[30] Cotten CM , Taylor S , Stoll B , et al. Prolonged duration of initial empirical antibiotic treatment is associated with increased rates of necrotizing enterocolitis and death for extremely low birth weight infants. Pediatrics. 2009;123:58‐66. 19117861 10.1542/peds.2007-3423 PMC2760222

[31] Abdel Ghany EA , Ali AA . Empirical antibiotic treatment and the risk of necrotizing enterocolitis and death in very low birth weight neonates. Ann Saudi Med. 2012;32:521‐526. 22871623 10.5144/0256-4947.2012.521 PMC6080994

[32] Kuppala VS , Meinzen‐Derr J , Morrow AL , Schibler KR . Prolonged initial empirical antibiotic treatment is associated with adverse outcomes in premature infants. J Pediatr. 2011;159:720‐725. 21784435 10.1016/j.jpeds.2011.05.033 PMC3193552

[33] Alexander VN , Northrup V , Bizzarro MJ . Antibiotic exposure in the newborn intensive care unit and the risk of necrotizing enterocolitis. J Pediatr. 2011;159:392‐397. 21489560 10.1016/j.jpeds.2011.02.035 PMC3137655

[34] Esaiassen E , Fjalstad JW , Juvet LK , van den Anker JN , Klingenberg C . Antibiotic exposure in neonates and early adverse outcomes: a systematic review and meta‐analysis. J Antimicrob Chemother. 2017;72:1858‐1870. 28369594 10.1093/jac/dkx088

[35] Krediet T , van Lelyveld N , Vijlbrief D , et al. Microbiological factors associated with neonatal necrotizing enterocolitis: protective effect of early antibiotic treatment. Acta Paediatr. 2003;92:1180‐1182. 14632335

[36] Jensen ML , Thymann T , Cilieborg MS , et al. Antibiotics modulate intestinal immunity and prevent necrotizing enterocolitis in preterm neonatal piglets. Am J Physiol Gastrointest Liver Physiol. 2014;306:G59‐G71. 24157972 10.1152/ajpgi.00213.2013 PMC4073901

[37] Berkhout DJC , Klaassen P , Niemarkt HJ , et al. Risk factors for necrotizing enterocolitis: a prospective multicenter case‐control study. Neonatology. 2018;114(3):277‐284. 29996136 10.1159/000489677

[38] Mokrohisky ST , Levine RL , Blumhagen JD , Wesenberg RL , Simmons MA . Low positioning of umbilical‐artery catheters increases associated complications in newborn infants. N Engl J Med. 1978;299:561‐564. 683224 10.1056/NEJM197809142991101

[39] Wesstrom G , Finnstrom O , Stenport G , Tyson JE , deSa DJ , Moore S . Umbilical artery catheterization in newborns. Acta Paediatr. 1979;68:575‐581. 10.1111/j.1651-2227.1979.tb05058.x 463541

[40] Tyson JE , deSa DJ , Moore S . Thromboatheromatous complications of umbilical arterial catheterization in the newborn period. Arch Dis Child. 1976;51:744‐754. 1008579 10.1136/adc.51.10.744 PMC1546133

[41] Roll C , Hanssler L . Effect of umbilical arterial catheters on intestinal blood supply. Acta Paediatr. 1998;87:955‐959. 9764890 10.1080/080352598750031617

[42] Shah JB , Bracero LA , Gewitz MH , Fish BG , Dweck HS . Umbilical artery catheters and blood flow velocities in the superior mesenteric artery: effect of insertion, removal, aspiration, and bolus infusion. J Clin Ultrasound. 1998;26:73‐77. 9460634 10.1002/(sici)1097-0096(199802)26:2<73::aid-jcu4>3.0.co;2-f

[43] Sulemanji MN , Azpurua H , Suh M , et al. Ductus venosus closure results in transient portal hypertension—is this the silent trigger for necrotizing enterocolitis? J Pediatr Surg. 2013;48:2067‐2074. 24094959 10.1016/j.jpedsurg.2013.01.022

[44] Coombs RC , Morgan MEI , Durbin GM , et al. Abnormal gut blood flow velocities in neonates at risk of necrotising enterocolitis. J Pediatr Gastroenterol Nutr. 1992;15:13‐19. 1403445 10.1097/00005176-199207000-00003

[45] Kempley ST , Gamsu HR . Superior mesenteric artery blood flow velocity in necrotising enterocolitis. Arch Dis Child. 1992;67:793‐796. 1519977 10.1136/adc.67.7_spec_no.793 PMC1590399

[46] Pettker CM , Buhimschi IA , Magloire LK , Sfakianaki AK , Hamar BD , Buhimschi CS . Value of placental microbial evaluation in diagnosing intra‐amniotic infection. Obstet Gynecol. 2007;109(3):739‐749. 17329528 10.1097/01.AOG.0000255663.47512.23

[47] Smulian JC , Shen‐Schwarz S , Vintzileos AM , Lake MF , Ananth CV . Clinical chorioamnionitis and histologic placental inflammation. Obstet Gynecol. 1999;94:1000‐1005. 10576190 10.1016/s0029-7844(99)00416-0

