# Supplementary material for: Umbilical arterial catheter duration as risk factor for Bell's Stage III necrotizing enterocolitis in preterm neonates
Source: JPGN Rep. 2024 May 20;5(3):256–64. doi: 10.1002/jpr3.12081 (PMC11322019; doi:10.1002/jpr3.12081)
Supplement: Supplementary file 1 — Supplementary Appendix S1: Modified Bell staging criteria for necrotizing enterocolitis. Supplementary Appendix S2: Calculation of Vasoactive Inotrope score (VIS). [file JPR3-5-256-s001.docx]

**Supplementary Appendix S1: Modified Bell staging criteria for necrotizing enterocolitis**

| **Stage** | **Classification<** | **Systemic Signs** | **Intestinal Signs** | **Radiologic Signs** |
| --- | --- | --- | --- | --- |
|  |  |  |  |  |
| IA | Suspected NEC | Temperature instability, apnea, bradycardia, lethargy | Increased pregavage residuals, midabdominal distention, emesis, guaiac-positive stool | Normal or intestinal dilation, mild ileus |
|  |  |  |  |  |
| IB | Suspected NEC | Same as above | Bright red blood from rectum | Same as above |
|  |  |  |  |  |
| IIA | Proven NEC—mildly ill | Same as above | Same as above, plus absent bowel sounds, with or without abdominal tenderness | Intestinal dilation, ileus, pneumatosis intestinalis |
|  |  |  |  |  |
| IIB | Proven NEC—moderately ill | Same as above, plus mild metabolic acidosis and mild thrombocytopenia | Same as above, plus absent bowel sounds, definite tenderness, with or without abdominal cellulitis or right lower quadrant mass | Same as IIB, plus definite ascites |
|  |  |  |  |  |
| IIIA | Advanced NEC—severely ill, bowel intact | Same as IIB, plus hypotension bradycardia, severe apnea, combined respiratory and metabolic acidosis, disseminated intravascular coagulation, and neutropenia | Same as above, plus signs of generalized peritonitis, marked tenderness, and distention of abdomen | Same as IIB, plus definite ascites |
|  |  |  |  |  |
| IIIB | Advanced NEC—severely ill, bowel perforated | Same as IIIA | Same as IIIA | Same as IIB, plus pneumoperitoneum |
|  |  |  |  |  |
| NEC = necrotizing enterocolitis. | | | | |

Neu J. Necrotizing enterocolitis: the search for a unifying pathogenic theory leading to prevention. Pediatr Clin North Am. 1996 Apr;43(2):409-32 (2)

**Supplementary Appendix S2: Calculation of Vasoactive Inotrope score (VIS)**

The VIS is calculated in the following manner:

VIS = dopamine dose (mg/kg/min) + dobutamine dose (mg/kg/min) + 100 x epinephrine dose (mg/kg/min) + 10 x milrinone dose (mg/kg/min) + 10,000 x vasopressin dose

(U/kg/min) + 100 x norepinephrine dose (mg/kg/min)
